# Comparative analysis of physiological and psychological effects of viewing and drinking flower tea

**DOI:** 10.1038/s41598-026-38690-6

**Published:** 2026-03-21

**Authors:** Chorong Song, Juhyeon Kim, Choyun Kim

**Affiliations:** Department of Forest Science, Kongju National University, 54 Daehak-ro, Yesan-eup, Yesan-gun, 32439 Chungcheongnam-do Korea

**Keywords:** Sensory experience, Emotional well-being, Relaxation effects, Herbal beverage, Autonomic response, Stress relief, Quality of life, Therapeutics

## Abstract

Flower tea has gained attention for its potential health benefits, yet empirical evidence regarding its comprehensive effects on human physiology and psychology remains limited. This study aimed to investigate how both visual stimulation by flowers and the act of drinking flower tea influence physiological and psychological relaxation. Twenty-nine university students (mean age: 21.0 ± 2.0 years) participated in a within-subject experimental design consisting of four stages: (1) resting with their eyes closed for 1 min (rest (before) period), (2) viewing tea with flowers, tea without flowers, or water (control) for 1 min after opening their eyes (viewing period), (3) drinking it for 3 min (drinking period), (4) closing their eyes again and resting for 1 min (rest (after) period), (5) responding to the questionnaire, and (6) taking a break for 5 min. After completing this sequence, the same process was repeated using a different stimulus. Heart rate variability and heart rate were measured as physiological indicators, while the Profile of Mood States and semantic differential scales were used as psychological indicators. Results showed that viewing tea with flowers significantly enhanced parasympathetic nervous system activity compared to other stimuli. Further, tea with and without flowers enhanced vigor alleviated total mood disturbances, and induced positive feelings throughout the entire process. These findings demonstrate that the combined visual and olfactory–gustatory experience of flower tea promotes physiological and psychological relaxation, suggesting its potential as an effective everyday method for stress relief and emotional well-being.

## Introduction

In today’s complex and competitive society, individuals are increasingly exposed to psychological stress and seek environments that promote comfort and relaxation. Among such environments, nature has been scientifically proven to enhance human health and well-being through its restorative and stress-reducing effects^1–10^. Nature therapy, a health-promotion method that uses medically proven effects, such as relaxation by exposure to nature^1,11^ has gained attention as a non-pharmacological approach for improving both mental and physical balance. Even when direct contact with nature is not feasible, indirect exposure through visual, olfactory, and auditory elements such as photographs, scents, and sounds has been shown to effectively reduce stress and induce physiological relaxation^12–17^. These findings highlight the growing relevance of nature-based sensory experiences as accessible and beneficial tools for stress management in modern daily life.

Previous studies have demonstrated that natural stimuli engage multiple sensory pathways to promote relaxation. Visual stimuli, such as photographs, 3D images, virtual reality, and videos of forests, lead to physiological relaxation responses, including decreased blood pressure, reduced sympathetic nerve activity, and calming brain activity^12^. Olfactory stimuli, including essential oils extracted from flowers and leaves, have been reported to rejuvenate the body^13^, calm brain activity, activate parasympathetic nerve responses, and reduce anxiety while improving sleep and mood states in middle-aged women^14,15^. Auditory stimuli, such as nature sounds and soundscapes, also contribute to improved mood, reduced arousal, and enhanced cognitive performance^16,17^. Collectively, these results demonstrate that multi-sensory stimulation derived from forest environments can promote both physiological and psychological restoration in humans.

In addition to these sensory elements, taste-related natural resources, such as fruits, herbs, and flowers, also play a role in stimulating human senses. Recently, flower tea (a beverage made by infusing edible flowers) has received increasing attention for its mind-soothing, anti-aging, and medicinal effects^18–21^. Flower tea is characterized by the harmonious combination of color, fragrance, and flavor, providing a unique multi-sensory experience that aligns well with nature therapy practices such as forest therapy, horticultural therapy, and healing agriculture. In forest therapy programs, tea-related activities are frequently used to promote relaxation and stress relief, yielding high participant satisfaction^22–24^. Furthermore, flower tea is recognized as a form of complementary and alternative therapy and classified as phytotherapy according to the U.S. National Center for Complementary and Alternative Medicine (NCCAM)^25–28^.

Building upon these findings, the effects of flower tea are determined through two complex mechanisms: (1) biochemical absorption of the tea’s active components after ingestion^29–31^, and (2) sensory stimulation through visual, olfactory, and gustatory engagement^32,33^. Although these mechanisms have been individually discussed in food science and aromatherapy research, the theoretical understanding of how multiple sensory inputs interact to influence human relaxation remains limited. In particular, few studies have explored the integrative framework of multisensory stimulation—a key concept in horticultural and nature-based therapy. This framework emphasizes the combined effects of visual, olfactory, and tactile experiences on physiological and emotional responses. While several studies have examined the chemical composition and health-related functions of edible flowers^34–36^, few have investigated how these properties translate into combined sensory experiences. Consequently, research on the integrated effects of viewing and drinking flower tea remains scarce. Most previous works have focused on either the pharmacological effects of flower compounds or the psychological responses to visual stimuli alone, leaving the interactive influence of these elements largely untested. Therefore, the specific role of visual perception during the drinking process—how it modulates the overall relaxation effect—remains an unresolved scientific question in the field of horticultural therapy. For example, Lee et al.^37^ demonstrated that horticultural activities and flower tea drinking improved the cognitive and emotional functions of older adults. Xue et al.^38^ examined that drinking magnolia tea for three weeks can ameliorate sleep quality and alleviate symptoms of depression in postpartum women. However, no studies have systematically investigated the unique impact of visual elements during flower tea consumption, leaving an important research gap regarding how combined sensory factors influence human physiological and psychological relaxation.

Therefore, this study aimed to comprehensively examine the physiological and psychological effects of both viewing and drinking flower tea. Specifically, we investigated how visual stimulation by flowers affects the drinking process and compared participants’ responses under three conditions: (1) tea with flowers (visual + olfactory + gustatory stimulation), (2) tea without flowers (olfactory + gustatory stimulation), and (3) water (control). Physiological indicators (including heart rate variability and heart rate) and psychological indicators (including mood state and emotional evaluation) were assessed. This study is the first to isolate and directly compare the visual effects of flower tea with its gustatory and olfactory components using both physiological and psychological indicators. By clarifying the role of visual elements in flower tea consumption, this study provides new insights into sensory-based relaxation methods which can be easily applied to enhance emotional well-being and stress relief in everyday life. The results are expected to contribute to the practical development of everyday nature-therapy practices, such as wellness programs and stress-relief beverages.

## Materials and methods

### Participants

This study included 29 male and female university students in their 20 s (Table 1). Because this study involved human participants, approval was obtained from the Institutional Ethics Committee (IRB no: KNU_IRB_2023-045) before proceeding with the experiment, and the study was registered with the Clinical Research Information Service (KCT0009470).

**Table 1:** Participant details.

Parameter	Total	Male	Female
N	29	12	17
Age (years)	21.0 ± 2.0	22.3 ± 1.7	20.0 ± 1.5
Height (cm)	166.8 ± 9.1	175.3 ± 4.9	160.8 ± 5.9
Weight (kg)	65.4 ± 14.6	71.7 ± 10.9	60.9 ± 15.6
Body mass index (kg/m^2^)	23.5 ± 5.2	23.3 ± 2.8	23.7 ± 6.5

This study recruited university students because they represent an age group commonly exposed to psychological stress and lifestyle imbalance. Thus, they were considered an appropriate population for evaluating the relaxation effects of flower tea. In addition, university students were selected because they were readily accessible and capable of following experimental instructions under controlled laboratory conditions. Although the sample size was limited, a priori power analysis using G*Power 3.1 confirmed that the number of participants (n = 24–29) was sufficient to achieve the statistical power required.

The inclusion criteria for selecting participants healthy university students aged 18 years or older and individuals who understood the purpose of the study and voluntarily agreed to participate by submitting written consent. The exclusion criteria included individuals currently receiving medical treatment, those with a history of allergic reactions or multiple drug side effects, those with heart disease, and those with abnormalities in vision, taste, or smell.

Recruitment was conducted by posting announcements that described the study’s purpose, procedure, schedule, measurement indicators, and compensation on university bulletin boards and community forums. Participants who expressed a voluntary interest in participating and met the selection criteria were recruited on a first-come-first-served basis. The selected participants were provided with information about the experiment date and location and were instructed to abstain from caffeine and alcohol consumption for two hours before participation.

On the day of the experiment, participants who arrived at the site received a detailed explanation of the purpose, content, procedures, and safety precautions. The participants confirmed their understanding of the study and voluntarily participated by signing a written informed consent form. They also provided informed consent for the publication of identifying information/images in an online open-access publication (e.g., presentation at academic conferences, publication of a paper, etc.). All methods were performed in accordance with the relevant guidelines of the Declaration of Helsinki.

### Experimental design and procedure

This study adopted a within-subject experimental design, in which all participants were exposed to all stimuli. Each participant completed the experiment individually under identical environmental conditions.

**Fig. 1:**
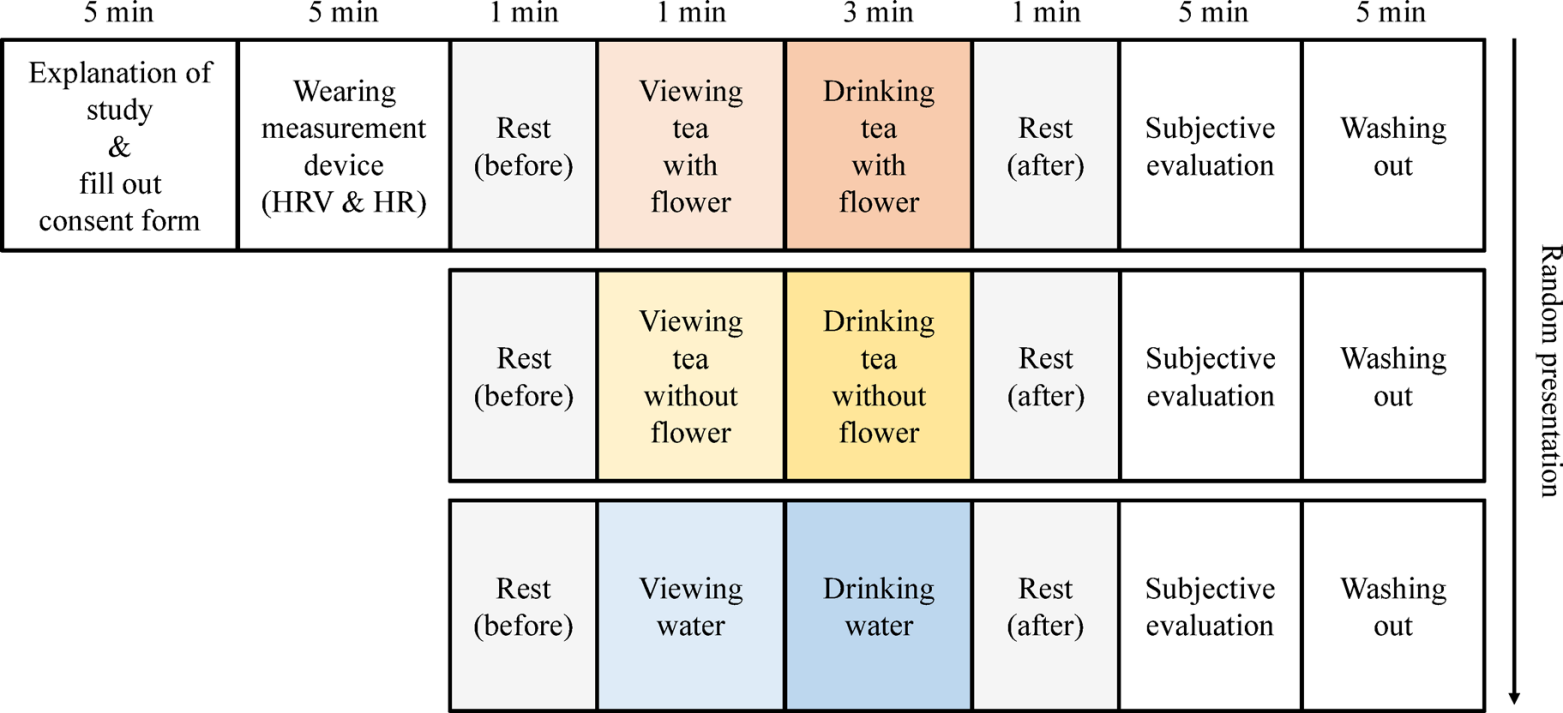
Experimental protocol.

**Fig. 2:**
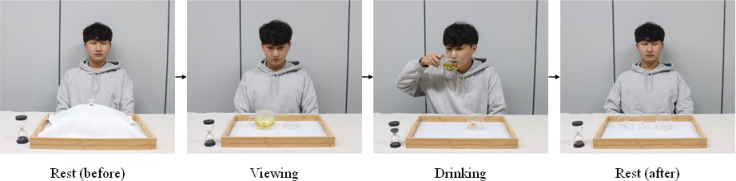
Experimental process and its representative images.

The overall procedure is illustrated in Fig. 1. The participants listened to an explanation of the experiment and filled out a consent form. Following the experimenter’s instructions, the participants were fitted with a physiological measurement device, seated on a chair, and allowed to wait momentarily in a relaxed state. Measurements were taken after approximately 5 min of setting up the experiment. The participants closed their eyes and rested for a minute (rest (before) period), after which they opened their eyes and viewed water (control), tea without flowers (taste stimulation), or tea with flowers (taste + visual stimulation) for a minute (viewing period). They then turned the hourglass (3 min) set on the desk and drank slowly at this pace (drinking period); this was followed by closing their eyes and resting again for a minute (rest (after) period). Images representing each stage of rest (before), viewing, drinking, and rest (after) are shown in Fig. 2.

After the rest (after) period, participants recalled their impressions during the viewing and drinking stages and completed the psychological questionnaires. After a 5 min break, the same procedure was repeated twice with different stimuli. The order of stimuli was randomized for each participant to minimize order effects.

### Stimuli (flower tea preparation)

*Kobus magnolia* was used as the flower tea as this tea met all of the following conditions: (1) it is registered for use in the Food Code (Notification No. 2021-26 as of March 25, 2021) by the Ministry of Food and Drug Safety in South Korea, (2) it is a widely consumed flower tea to South Korea^39^, (3) it is the flower of a woody plant native, and (4) the tea retains the complete form of the flower (petals, stamens, etc.).

Magnolia flowers used for the tea were purchased as a commercially available product. A 300 ml round glass cup was used with a strainer of the same material. After viewing the tea or water, participants removed the strainer and placed it on the lid that the experimenter had previously set on the left side of the cup, then drank the tea (see viewing and drinking in Fig. 2).

In this study, two 1.5 L electric pots were used. The same pot was used for both tea with and without flowers to ensure identical brewing times and concentrations, whereas a separate pot was used for water. Each pot was filled with 1 L of bottled water, heated simultaneously to 100 °C, and then kept warm at 70 °C.

The tea with and without flowers was prepared by adding three magnolia flowers when the water reached boiling point (100 °C) and the alarm sounded, and then steeping for more than 5 min before brewing. For tea with flowers, the most aesthetically pleasing flower among the brewed ones was placed on the tea strainer, and the steeped tea was poured over it. Only the steeped liquid was used for teas without flowers.

**Fig. 3:**
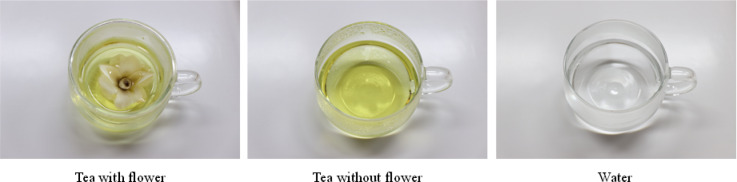
Visual and gustatory stimuli of tea with flower, tea without flower, and water.

The volume of all three stimuli was standardized to 150 ml (Fig. 3) and adjusted using an electronic scale and a dropper pipette.

Because temperature is a significant factor affecting the results^40,41^, we ensured that all participants drank the tea or water at the same temperature as much as possible. Tea was prepared 5 min before the rest (before) period (or during the washout time for the second stimulus) to ensure that the temperature at the time of appreciation and drinking for all three stimuli was as identical as possible.

During the experimental setup, the tray was covered and placed on a desk to prevent participants from knowing in advance what the next stimulus would be (refer to Fig. 2, rest (before)).

The temperature right before the participants drank the tea was approximately 55 °C, and when it was maintained until the end of the experiment without drinking it, it was approximately 50 °C.

Participants drank the set amount (150 ml) at their own pace, using the hourglass to check the time during the 3 min drinking period.

### Physiological measurements

#### Heart rate and heart rate variability

The heart rate and heart rate variability (HRV) were measured. Heart rate refers to the number of heartbeats per minute. HRV is an indicator of autonomic nerve activity, which reflects the tension and relaxation of the human body, and is a physiological measurement method widely used in the field of forest therapy^7^.

Heart rate variability can determine the degree of activation for parasympathetic nerve activity increased in the relaxed state, and sympathetic nerve activity increased in tense or stressful states, based on calculation of high-frequency and low-frequency components through frequency analysis after measuring the interval between heartbeats^42^.

We measured the intervals between consecutive R waves for each participant using a portable electrocardiogram (myBeat; Union Tool Co., Tokyo, Japan) and performed frequency analysis using MemCalc/Win software (GMS, Tokyo, Japan). The power levels of the low-frequency (LF; 0.04–0.15 Hz) and high-frequency (HF; 0.15–0.40 Hz) components of HRV were calculated using the maximum entropy method^43^. The HF component of HRV reflects parasympathetic nervous activity, and the LF/HF ratio reflects sympathetic nervous activity^44^. We used natural logarithmic-transformed values in the analysis to normalize the HRV parameters across participants^45^.

### Psychological measurements

#### Profile of mood state: K-POMS-B

The POMS questionnaire has been developed to assess mood states^46^. It comprises 30 items evaluated on a 5-point Likert scale, which in turn includes six subscales—Tension-Anxiety (T-A), Depression (D), Anger-Hostility (A-H), Fatigue (F), Confusion (C), and Vigor (V). The formula [(T-A) + (D) + (A-H) + (F) + (C)–(V)] was used to calculate the total mood disturbance (TMD). The Korean version of the POMS was used^47^.

#### Semantic differential (SD) method

The SD method evaluates the impressions of a subject using adjectives that express emotions^48^. It uses 27 pairs of opposing adjectives for evaluation on a 7-point Likert scale.

### Data analysis

Differences in participants’ responses were analyzed among three stimuli: (1) water (control), (2) tea without flower (gustatory + olfactory stimulation), and (3) tea with flower (gustatory + olfactory + visual stimulation). By comparing (1) and (2), we examined the effect of tea consumption, and by comparing (2) and (3), we identified the effect of adding visual floral elements.

Five participants were excluded from HRV analysis because of measurement errors; thus, data from 24 participants were used. A power analysis was conducted with G*Power 3.1 to determine the minimum sample size (effect size f = 0.25, α = 0.05, power = 0.70, repeated measures ANOVA, three measurements, correlation among measures = 0.5, nonsphericity correction = 1). The required minimum sample size was 24, satisfying this criterion.

Statistical analyses were performed using SPSS 26.0 (IBM Corp., Armonk, NY, USA) with significance set at p < 0.05. Baseline consistency for physiological indicators was verified using one-way ANOVA with rest (before) values as baselines. Because the psychological measurement was conducted immediately after each condition (~ 5 min), no separate pre-measurement was performed.

A one-way repeated-measures ANOVA with Bonferroni post-hoc test was applied for physiological indicators, and the Friedman test with Bonferroni correction was used for psychological indicators.

## Results

### Physiological responses

**Fig. 4:**
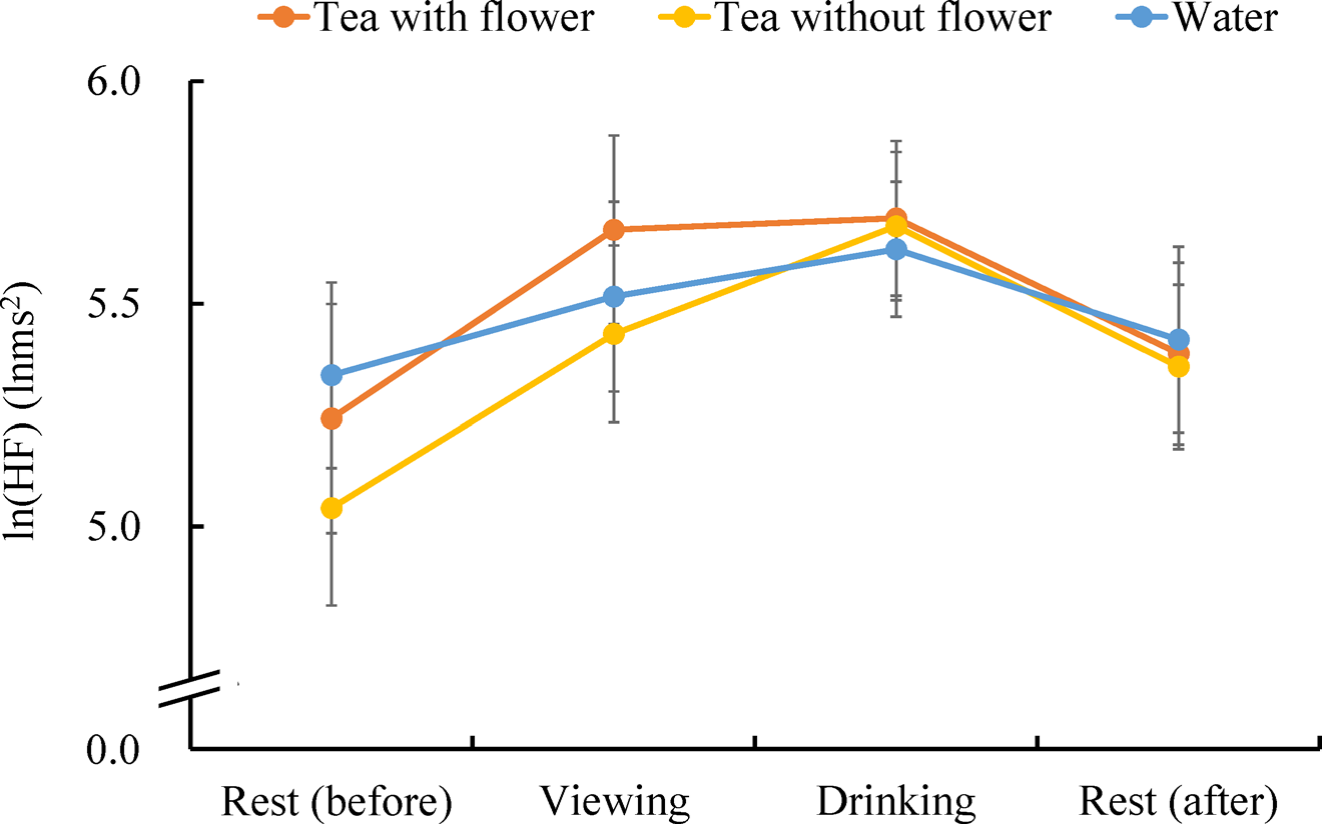
Changes in parasympathetic nervous activity (lnHF) across experimental phases.

Figure 4 presents the ln(HF) values obtained across the entire period. The ln(HF) values increased for all stimuli when transitioning from the rest (before) period to the viewing period and from the viewing period to the drinking period. Additionally, ln(HF) decreased during the rest (after) period after the drinking period.

We analyzed the differences among tea with flowers, tea without flowers, and water during each period. Initially comparing the three stimuli in the rest (before) period yielded no significant differences (tea with flower: 5.24 ± 0.26 lnms^2^, tea without flower: 5.04 ± 0.22 lnms^2^, water: 5.34 ± 0.21 lnms^2^, P > 0.05), indicating that there were no differences in the stable state before the stimuli.

**Fig. 5:**
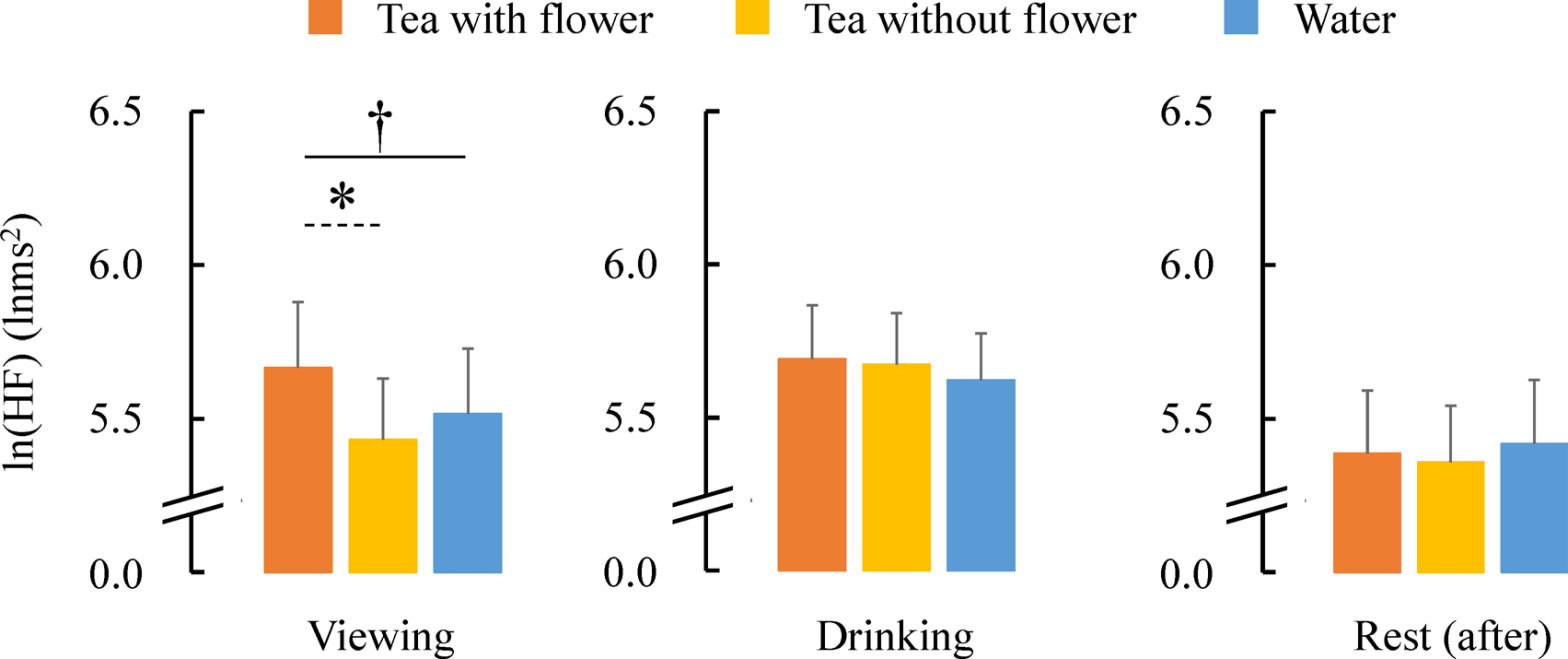
Parasympathetic nervous activity (lnHF) under three beverage conditions during viewing, drinking, and rest phases. N = 24, mean ± standard error, †: p < 0.05 by one-way repeated measured ANOVA, *: p < 0.05 by Bonferroni correction (post-hoc test).

Figure 5 presents the differences among the three stimuli during the viewing, drinking, and rest (after) periods. Significant differences were observed among the three stimuli during the viewing period (tea with flower: 5.67 ± 0.21 lnms^2^, tea without flower: 5.43 ± 0.20 lnms^2^, water: 5.52 ± 0.21 lnms^2^, p < 0.05), and the post-hoc tests revealed a significant difference between the values obtained for tea with flower and tea without flower. The ln(HF) value, an index of parasympathetic nervous system activity that becomes activated during a relaxed state, increased further when flowers were present. However, no significant differences were observed in both the drinking period (tea with flower: 5.69 ± 0.17 lnms^2^, tea without flower: 5.67 ± 0.17 lnms^2^, water: 5.62 ± 0.15 lnms^2^) and the rest (after) period (tea with flower: 5.39 ± 0.20 lnms^2^, tea without flower: 5.36 ± 0.18 lnms^2^, water: 5.42 ± 0.21 lnms^2^).

No significant differences were determined in the ln(LF/HF) value, which is an index of sympathetic nervous system activity, or in the heart rate.

### Psychological responeses

**Fig. 6:**
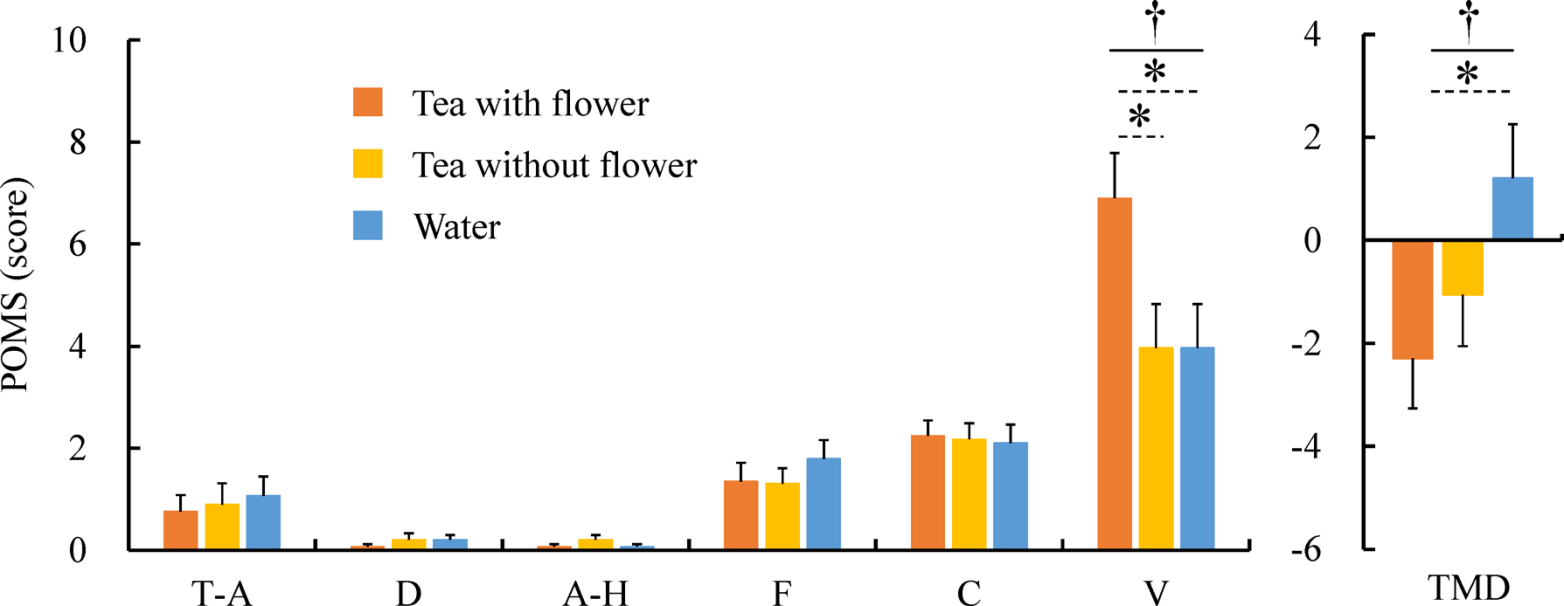
Changes in mood states after viewing and drinking flower tea. N = 29, mean ± standard error, †: p < 0.05 by Friedman test, *: p < 0.05 by Bonferroni correction (post-hoc test).

Analysis of the POMS revealed a significant difference in the positive mood based on the V subscale and TMD (Fig. 6). The scores for the V subscale were 6.9 ± 0.9 for tea with flower, 4.0 ± 0.9 for tea without flower, and 4.0 ± 0.9 for water, indicating a significant difference among the three stimuli (p < 0.05). The post hoc tests revealed significant differences between the tea with flower and tea without flower, as well as between tea with flowers and water, indicating that tea with flower elicited a more invigorating mood than that with the stimuli. Further, the TMD scores were –2.4 ± 1.1 for tea with flower, –1.2 ± 1.2 for tea without flower, and 1.3 ± 1.2 for water, indicating significant differences among the three stimuli (p < 0.05). Post hoc tests revealed a significant difference between tea with flowers and water, indicating that tea with flowers reduced the total mood disturbance more than water.

**Fig. 7:**
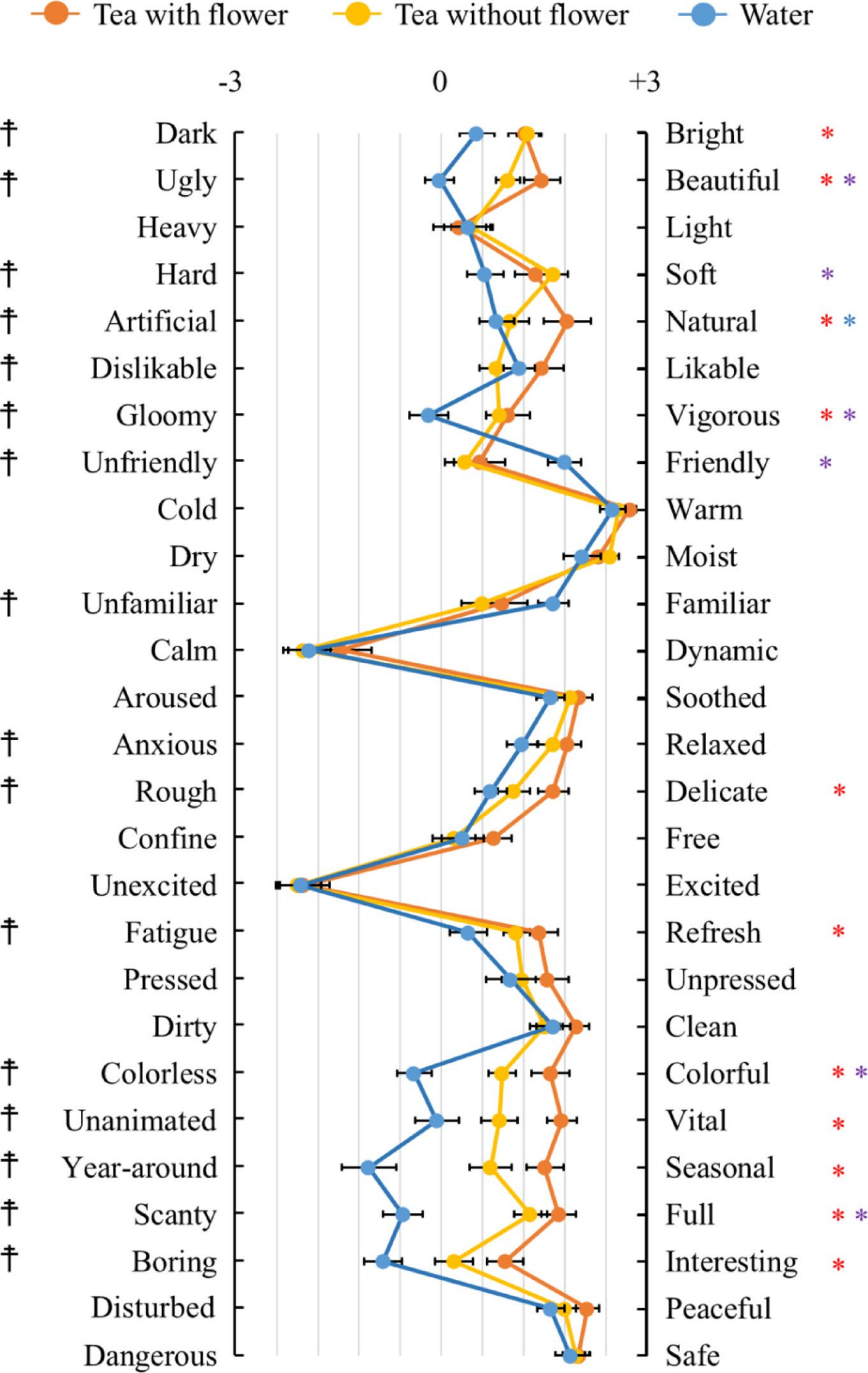
Emotional impressions evaluated by the Semantic Differential (SD) method. N = 29, mean ± standard error, †: p < 0.05 by Friedman test, *: p < 0.05 by Bonferroni correction (post-hoc test; red: tea with flower vs. water; purple: tea without flower vs. water; blue: tea with vs. without flower).

Figure 7 illustrates the results of the semantic differential (SD) analysis. Significant differences were observed in 16 pairs of adjectives (bright-dark, beautiful-ugly, soft-hard, natural-artificial, likable-dislikable, vigorous-gloomy, friendly-unfriendly, familiar-unfamiliar, relaxed-anxious, delicate-rough, refresh-fatigue, colorful-colorless, vital-unanimated, seasonal-year round, full-scanty, and interesting-boring) at a significance level of p < 0.05. Tea with flowers was found to evoke more feelings of bright, beautiful, natural, vigorous, relaxed, delicate, refresh, colorful, vital, seasonal, full, and interesting compared to those evoked with water. Tea without flower was perceived to elicit more feelings of beautiful, soft, vigorous, friendly, colorful, and full compared to those with water. Furthermore, tea with flowers imparted a greater feeling of naturalness than that with Tea without flower.

## Discussion

This study aimed to elucidate the physiological and psychological effects of viewing and drinking flower tea on human participants. By examining the effects of incorporating the visual stimulus of flowers into the process of drinking—combining the consumption of flower tea ingredients with gustatory and olfactory stimulation—this study comprehensively revealed the overall impact of flower tea.

First, based on heart rate variability analysis, viewing flower tea was associated with increased parasympathetic nervous system activity, suggesting a relaxation response. This finding aligns with previous studies that elucidated human responses when viewing natural elements, including flowers^12,49–52^. These studies have reported that visual stimuli from plants, such as flowers, foliage plants, or bonsai, activate the parasympathetic nervous system while suppressing sympathetic nervous system activity. Therefore, the visual components of flower tea play significant roles in physiological relaxation.

However, no significant differences were observed among the three stimuli during the drinking and rest (after) periods, suggesting that the act of drinking itself, rather than the type of beverage, may have had a stronger influence on autonomic responses. During the drinking period, the ln(HF) for tea with flower and tea without flower was slightly higher than that for water, but the difference was minimal. This indicates that the physical process of drinking, including swallowing movements, may have directly affected autonomic nervous system activity. Specifically, during the measurement of heart rate variability, water consumption was considered a potential confounding factor, and efforts were made to control for its effects^53^. Watanabe et al.^54^ investigated the correlation between the power ratio, which reflects stomach activity, and heart rate variability before and after drinking 150 ml of water. The results revealed a positive correlation between the rates of change (post-intake/pre-intake) in the power ratio and HF, indicating that increased stomach activity from water intake was associated with higher HF values. The current findings are therefore consistent with those previous results. This study aimed to determine whether differences arose from the three stimuli when the quantity and duration of water and tea consumption were identical; However, the act of consumption itself had a greater impact on heart rate variability than the taste and scent of tea. During the rest (after) period, no significant difference was observed because the process of offsetting the influence of drinking was reflected.

At the psychological level, however, significant results were obtained from the subjective evaluations conducted after drinking tea with flowers. POMS analysis presented a significant increase in vigor, resulting in a decrease in the total mood disturbance. These findings indicate that drinking flower tea induced psychological relaxation, as positive mood states increased while emotional disturbances were alleviated. Further, SD analysis revealed that the flower tea evoked feelings of bright, beautiful, natural, soft, vigorous, relaxed, delicate, refresh, friendly, colorful, vital, seasonal, full, and interesting. These results are consistent with previous studies on the psychological effects of various natural stimuli on humans^55–59^. A comprehensive examination of the results of the POMS and SD analyses in this study suggests that flower tea enhances psychological well-being by inducing positive feelings rather than by merely reducing negative feelings.

A significant difference was found in the ln(HF) in HRV, but no significant difference was found in ln(LF/HF), which may be interpreted in a similar context. Comfort can be classified into passive and active^60^. Passive comfort is based on the need for deficiency and aims to eliminate discomfort, whereas active comfort is a desire for growth by appropriate stimulation and aims to acquire positive α^60^. In modern society, active comfort is emphasized more preferentially. Indeed, the significance of these effects of flower tea holds substantial value in contemporary society, and it is believed to facilitate stress relief and psychological relaxation in daily life.

In addition, although the physiological responses observed in this study were modest in magnitude, they consistently reflected parasympathetic activation and improvements in mood states. These findings suggest that even short-term engagement with flower tea can induce measurable relaxation. Importantly, this study provides preliminary scientific evidence supporting the use of flower tea as a simple, accessible means to promote emotional well-being in daily life. In this context, flower tea may serve as a practical component of therapeutic tea art programs or nature-based relaxation activities, particularly for socially stressed groups such as university students. Future studies should expand on these findings through long-term and field-based applications.

Taken together, these findings demonstrate that flower tea can offer both physiological and psychological relaxation benefits, supporting its potential as a sensory-based approach to stress management.

Although this is the first comprehensive study to elucidate the physiological and psychological effects of flower tea on the human body, it also has several limitations. First, the focus of this study was solely on visual and taste stimuli, and we could not entirely control olfactory stimuli. Although magnolia flower tea is not heavily scented, olfactory stimuli may have had an effect. This limitation was unavoidable because olfactory control could interfere with the natural drinking experience; however, the potential influence of scent was minimized by using a tea with a relatively mild fragrance. Future studies are encouraged to examine and control olfactory factors more systematically. Second, this study was conducted on a single-flower tea, magnolia. This was an intentional design choice to minimize variation in sensory properties and isolate the visual effect of the flower. In the future, it is necessary to continuously accumulate research data by verifying the effects on various flower teas. Third, the study participants were limited to male and female university students in their twenties. Thus, participants with diverse attributes should be included in the future, and continuous accumulation of research data is needed to elucidate the effects of flower tea. While we acknowledge that this relatively homogeneous sample limits the generalizability of the findings, it nonetheless provides valuable baseline data for future studies involving participants from more diverse backgrounds. Finally, we could only demonstrate the effects over a short duration: 1 min of viewing, 3 min of drinking, and 1 min of rest (after). The short exposure period was chosen to evaluate immediate physiological and psychological responses under controlled conditions. Despite the brief duration, significant changes were observed, indicating that even short-term exposure can induce relaxation. Nevertheless, future studies should investigate the effects of longer or repeated exposure to clarify the sustainability of the response.

## Conclusions

This study aimed to elucidate the physiological and psychological effects of viewing and drinking flower tea on the human body. Verification through evaluation of heart rate variability, POMS, and SD revealed that viewing flower tea activated the parasympathetic nervous system in the human body. Additionally, the entire period of viewing, drinking, and rest (after) was found to enhance vigor, reduce mood disturbances, and induce a positive mood. In conclusion, viewing and drinking flower tea can exert positive effects on physiological and psychological relaxation.

## Data Availability

The datasets generated and/or analyzed during the current study are not publicly available but are available from the corresponding author on reasonable request.
